# Acoustic Emission Signal Fault Diagnosis Based on Compressed Sensing for RV Reducer

**DOI:** 10.3390/s22072641

**Published:** 2022-03-30

**Authors:** Jianwei Yang, Chang Liu, Qitong Xu, Jinyi Tai

**Affiliations:** School of Key Laboratory of Vibration and Noise under Ministry of Education of Yunnan Province, Kunming University of Science and Technology, Kunming 650500, China; yjw326101@163.com (J.Y.); xqt3163373908@163.com (Q.X.); tjy1320828693@163.com (J.T.)

**Keywords:** RV reducer, compressed sensing, acoustic emission signal, multi-channel convolutional layer, energy pooling layer

## Abstract

The rotate vector (RV) reducer has a complex structure and highly coupled internal components. Acoustic emission (AE) signal, which is more sensitive to a weak fault, is selected for fault diagnosis of the RV reducer. The high sampling frequency and big data are the challenges for AE signal store and analysis. This study combines compressed sensing (CS) and convolutional neural networks. As a result, data redundancy is significantly reduced while retaining most of the information, and the analysis efficiency is improved. Firstly, the time-domain AE signal was projected into the compression domain to obtain the compression signal; then, the wavelet packet decomposition in the compressed domain was performed to obtain the information of each frequency band. Next, the frequency band information was sent into the input layer of the multi-channel convolutional layer, and the energy pooling layer mines the energy characteristics of each frequency band. Finally, the softmax classifier was used to classify and predict different fault types of RV reducers. The self-fabricated RV reducer experimental platform was used to verify the proposed method. The experimental results show that the proposed method can effectively extract the fault features in the AE signal of the RV reducer, improve the efficiency of signal processing and analysis, and achieve the accurate classification of RV reducer faults.

## 1. Introduction

With the rapid development of the manufacturing industry, industrial robots have become the core execution unit of intelligent manufacturing. The RV reducer is the core component of industrial robots, and its health status is the main reason to limit the long-term stable operation of industrial robots [1,2]. Therefore, it is very important to study the fault diagnosis of RV reducers. The structure of RV reducers is very complex, with special structure and dynamic characteristics, which also leads to its complex dynamic response, thus increasing the frequency spectrum complexity of the vibration signal [3,4]. For weak fault signals, the characteristics of fault signals are very weak and easily immersed in the noise of other components, so they cannot be effectively diagnosed [5]. AE refers to the phenomenon that materials are deformed or fractured by external or internal forces, releasing stress-strain in the form of elastic waves [6]. AE technology can be used for non-destructive testing of materials or structures with damage or initial damage. AE technology can dynamically monitor the stress changes inside and on the surface of materials, which is an effective method for fault diagnosis [7]. Compared with the vibration signal analysis method, the AE signal is more sensitive, less affected by the mechanical background noise, and can diagnose weak faults. Therefore, the method of fault diagnosis based on the AE signal has been widely used [8,9]. However, AE signal acquisition requires a very high sampling frequency and generates a large amount of redundant data. This method not only requires high sampling equipment but also increases the difficulty of data storage, transmission, and analysis.

The emergence of CS theory provides a new solution to this problem. Donoh et al. [10,11] proposed a new signal acquisition and processing method based on sparse representation and signal approximation theory, namely CS. The signal can be sampled far below the Nyquist sampling theorem, and the original signal can be reconstructed with high probability. The original signal can be recovered from CS data. In this sense, the CS data contains sufficient information about the original signal. Part of the noise and redundant information in the signal can also be eliminated, which can greatly reduce the amount of collected data, reduce the pressure on the collection end, improve data transmission efficiency, and reduce data storage space. Therefore, the AE fault diagnosis method based on CS has received widespread attention. Guo et al. [12] compared the basic performance of different reconstruction algorithms of wavelet and discrete cosine transform (DCT) based on the theoretical basis of CS. Wang et al. [13] proposed a fault diagnosis method based on compressed sparse time-frequency feature representation of CS, which can reconstruct the time-frequency feature of fault signal from a small amount of compressed sampling data containing noise. Cheng et al. [14] proposed a method for health index extraction of planetary gearbox based on sparse representation and reconstruction theory. This method does not need to accurately recover the original signal, but only needs to diagnose the planetary gearbox based on some sparse representation and reconstruction results of redundant dictionaries of original signals. Liu et al. [15] proposed a method to directly extract the AE signal compression feature (AECF) from the CS data to evaluate the operating state of rotating machinery. However, the method of fault diagnosis of mechanical equipment through reconstruction algorithm and feature extraction from CS data relies on the signal processing and feature extraction experience of diagnostic experts, which will cause the loss of sensitive information in the data. This directly affects the accuracy of diagnostic results.

Deep learning can automatically learn representative features from data, which largely removes the experience of signal processing and feature extraction relying on diagnostic experts, and has been gradually applied to the field of fault diagnosis [16,17]. Chen et al. [18] proposed a fault state identification method of planetary gearbox based on convolution neural network and discrete wavelet transform for the non-stationary and nonlinear vibration signal of planetary gearbox. Peng et al. [19] proposed a noise deep convolution neural model (NOSCNN) to identify the faults of RV reducers under different working conditions. Sun et al. [20] adopted nonlinear projection to achieve compression acquisition, and established a depth neural network based on stacked sparse automatic encoder for fault identification of rotating machinery. Shao et al. [21] proposed a new method of improved convolution deep belief network (CDBN) based on CS. A new CDBN model was constructed with Gaussian visible units to enhance the feature learning ability for the CS data of mechanical equipment. Song et al. [22] used CS to improve the effectiveness of real-time performance of crack induced AE signal analysis in track structure health monitoring (SHM), and proposed a reconstruction method of multiscale-modular dictionary based on a multiscale dataset to improve the real-time performance of reconstruction. Hu et al. [23] proposed a data-driven fault diagnosis method based on CS and improved multiscale network (IMSN) to identify and classify faults in rotating machinery. In the above research, the deep neural network was used to mine the hidden fault characteristic information for the diagnosis of rotating machinery. However, the structure of the RV reducer is very complex, and the collected AE signals only represent the local characteristic information, which cannot represent all the nonlinear characteristics. Therefore, the combination of CS and deep learning model will make the deep learning model have a better automatic feature extraction ability for fault diagnosis of RV reducers. While CS theory will compress and sample the AE signal, which greatly reduces the amount of data redundancy while retaining most of the information, and improves the analysis efficiency. Combining with deep learning models will have more advantages.

In summary, this study uses the CS theory to compress the acquired AE signal to obtain compressed data, which solves the problems in which the sampling frequency of the AE signal is too high, and the amount of data is too large, which makes data storage, transmission, and analysis difficult. The time-domain AE signal was projected into the compression domain to obtain the compressed signal. The wavelet packet change matrix in the compression domain was used to decompose the compressed signal to obtain the information of each frequency band. The obtained information of each frequency band is the input signal of the multi-channel convolutional layer; the energy pooling layer was used to mine the energy characteristics of each frequency band. The proposed method was verified by a self-fabricated RV reducer experiment. The experimental results show that the method proposed in this study has great advantages in accuracy and robustness. The main contributions of this study include the following:

(1) The combination of CS technology and AE signal not only retains most of the effective information, but also greatly reduces the amount of data of AE signal;
(2) According to CS, the transform matrix of wavelet packet decomposition in compressed domain was derived, which was used to decompose the compressed signal, extracting the compressed domain signals of different frequency bands;
(3) The data layer fusion method based on multi-channel fusion convolutional neural network (MF-CNN) model takes the obtained frequency band information as the input signal of a multi-channel convolution layer, which can effectively mine the features of different frequency bands and avoid the uncertain of diagnosis results caused by subjectively selecting the features information of different frequency bands;
(4) The energy features of information are extracted through the energy pooling layer to improve the ability of one-dimensional convolutional neural network (1-DCNN) to explore the energy features of signal and fully mine the hidden features of data.

## 2. Theoretical Background

### 2.1. Compressed Sensing Theory

CS can simultaneously achieve signal compression and sampling at a sampling frequency less than twice the original signal frequency. CS theory consists of three parts: signal sparse representation, observation matrix design and signal reconstruction. Sparse representation is the premise of CS. Most signals in nature are not sparse signals, but a specific sparse base Ψ can be found to make the signal sparse. It is sparse to project the original signal $x\in R^{N\times 1}$ into a sparse base $\Psi \in R^{N\times N}$.

(1) x=Ψθ

where the transformation coefficient θ is sparse and contains only a small number of non-zero terms. The observation matrix Φ unrelated to Ψ is selected to reduce the dimension of the original signal to achieve data compression. Assuming the observation matrix $\Phi \in R^{M\times N}$, there is:

(2) y=Φx

The CS signal $y\in R^{M\times 1}$ is obtained. When y, Ψ, and Φ are known, according to the sparse property, the signal reconstruction is often realized by optimizing $l_{1}$-norm [24] and greedy algorithm.

### 2.2. Random Projection Energy Preservation Property

According to the CS theory, for signal $x\in R^{N\times 1}$, the observation matrix Φ was used as the projection matrix to compress the signal. The process of data compression is the process of linear projection according to the distance preserving property (DPP) condition of the projection matrix [25]. The random projection distance preserving of time domain signal x can be described as:

(3) $$\left(1-\varepsilon \right){\left\|x\right\|}_{2}^{2}\leq {\left\|\hat{x}\right\|}_{2}^{2}\leq \left(1+\varepsilon \right){\left\|x\right\|}_{2}^{2}$$

where ε∈(0,1), from the perspective of energy, the energy of the signal is approximately unchanged before and after projection. Accordingly, the energy feature parameter E of the time domain signal and the energy feature parameter $\hat{E}$ of the compressed domain signal are defined as:

(4) $$E={\left\|x\right\|}_{2}^{2}=\sum_{i=1}^{N}x_{i}^{2},\hat{E}={\left\|\hat{x}\right\|}_{2}^{2}=\sum_{i=1}^{N}{\hat{x}}_{i}^{2}$$

Random projection energy preservation can be described as:

(5) $$\left|\hat{E}-E\right|\leq C$$

where C is a constant with a very small value.

According to Equation (5), the signal energy approximately remains unchanged during the random projection process, that is, the energy feature $\hat{E}$ of the signal in the compressed domain and the energy feature E of the time-domain signal are approximately the same. Based on this property, in the process of time-domain signal analysis, the energy features of compressed data were analyzed and the diagnosis results are approximately consistent with the diagnosis results obtained from the energy features of the original signal in time domain.

### 2.3. Transformation Matrix of Wavelet Packet Decomposition in Compressed Domain

From the perspective of function theory, wavelet packet decomposition is to project the signal into the space expanded by wavelet packet basis function. From the perspective of signal processing, it enables the signal to decompose in different frequency bands, subdivides the frequency band into many levels and further subdivides the high-frequency portion of the band that is not subdivided by a wavelet analysis. According to the random time frequency resolution, the wavelet packet decomposes the signal into the corresponding frequency band components, which provides efficient and powerful results for the non-stationary description of dynamic signal, fault feature frequency, weak information extraction, and early fault diagnosis [26]. In this study, wavelet packet transform was used to decompose the compressed signal. The time-domain transformation matrix $H\in R^{N\times N}$ of the original signal $x\in R^{N\times 1}$ is defined, and the signal decomposition process is described as:

(6) f=Hx

where $f\in R^{N\times 1}$ is the signal component of the original signal x after spatial decomposition in time domain. According to the energy retention property of random projection, the compressed signal component $\hat{f}$ is obtained by projecting the original signal component f on the random measurement matrix Φ.

(7) $$\hat{f}=\Phi f$$

Similar to the signal transformation process in the time domain, the signal transformation matrix in the compressed domain is defined as $\hat{H}\in R^{M\times M}\left(M<N\right)$, and the decomposition process of the compressed domain signal $y\in R^{M\times 1}$ is described as:

(8) $$\hat{f}=\hat{H}y$$

According to Equations (6)–(8), the relationship between two different spatial transformation matrices H and $\hat{H}$ can be obtained as:

(9) $$\hat{f}=\Phi f=\Phi Hx=\hat{H}\Phi x$$

(10) $$\hat{H}=\Phi H\Phi^{-1}$$

According to Equation (10), the transformation matrix $\hat{H}$ of the wavelet packet decomposition in the compressed domain can be calculated, which defines the energy feature $E_{f}={\left\|f\right\|}_{2}^{2}=\sum_{i=1}^{N}f_{i}^{2}$ of the signal component f after the original signal x is decomposed in the time-domain space, and the energy feature ${\hat{E}}_{f}={\left\|\hat{f}\right\|}_{2}^{2}=\sum_{i=1}^{N}{\hat{f}}_{i}^{2}$ of the compressed signal component $\hat{f}$, according to the random projection energy retention properties available:

(11) $$\left|{\hat{E}}_{f}-E_{f}\right|\leq C_{f}$$

where $C_{f}$ is a constant with a small value. Therefore, on the premise of ensuring that the compressed signal retains enough effective information of the original signal, this study can obtain the information of each frequency band for the compressed signal as the input signal of the multi-channel convolution layer, effectively mine the features of different frequency bands, and then extract the information energy features through the energy pooling layer, and fully mine the hidden feature information of the data for diagnosis and analysis.

## 3. One-Dimensional Convolutional Neural Network (1-DCNN)

CNN was first applied to image recognition technology. It has the features of local connection, weight sharing and down-sampling, which greatly reduces the scale of the network structure, and can make full use of the local features of the data itself to improve the computing efficiency [27,28]. A typical CNN includes a convolution layer, pooling layer, full connected layer, and output layer [29]. The main difference between 1-DCNN and 2-DCNN is that the dimension of the feature graph is one-dimensional, so it is composed of a one-dimensional convolution layer, one-dimensional pooling layer, fully connected layer, and classifier.

### 3.1. Multi-Channel Fusion Convolutional Layer

Standard one-dimensional convolution layer: assume that one-dimensional signal $Y_{i}$ is the output of a feature graph of the i layer, and its convolution calculation method is:

(12) $$Y_{j}^{l}=f(\sum_{i\in M_{j}}(Y_{i}^{l-1}*w_{ij}^{l})+b_{j}^{l})$$

where $Y_{j}^{l}$ is the *j*th output of layer l; $M_{j}$ is the *j*th convolution region of layer l−1, and $Y_{i}^{l-1}$ is the *i*th feature input of the convolution layer of layer l−1; $w_{ij}^{l}$ is the corresponding convolution kernel; $b_{j}^{l}$ is the offset vector of the *l*th layer; f(x) is the activation function of the *l*th convolution layer.

**Figure 1:**
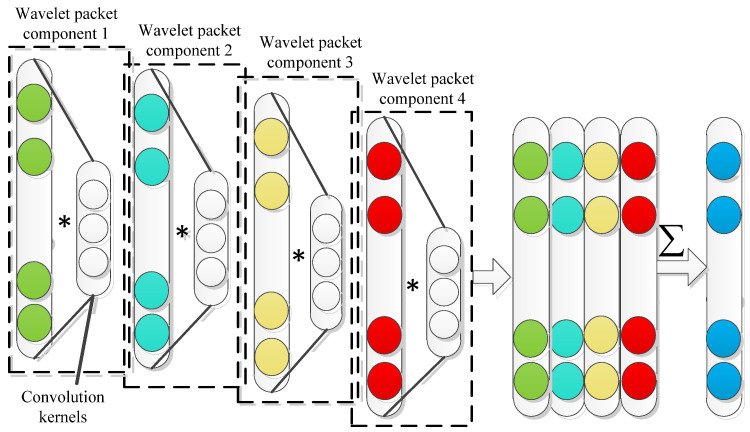
Multi-channel convolution kernel (∗ represents the convolution operation).

The compressed signal is decomposed by a wavelet packet in the compressed domain to obtain signal components of different frequency bands. Since different frequency bands contain local feature information of signals, if this feature information can be fully utilized, the accuracy of fault feature can be further improved. The obtained information of each frequency band was used as the input signal of the multi-channel convolution layer, which corresponds to the multi-channel convolution kernel. Different convolutions were applied to each channel data for each convolution operation. According to Equation (13), it can be regarded as weighting the signal features of different frequency bands after decomposition. The structure diagram of the fusion layer is shown in Figure 1.

(13) $$Y^{l}=\sum_{j=1}^{m}f_{j}(\sum_{i=1}^{k}(y_{ij}^{l-1}*w_{ij}^{l-1})+b_{j}^{l-1})$$

where $Y^{l}$ is the output of the *l*th convolutional layer; $y_{ij}^{l-1}$ is the *i*th feature input of the (l−1)th convolutional layer of channel j, with a total of k feature inputs; $w_{ij}$ is the size of the convolution kernel of the (l−1)th layer of channel j; $b_{ij}$ is the offset vector of the (l−1)th layer of channel j; $f_{j}(x)$ is the activation function of the (l−1)th convolution layer of channel j; m is the number of channels. The multi-channel convolution layer can automatically mine the fault feature information of signals in different frequency bands and can realize the adaptive selection of signal features in different frequency bands.

### 3.2. Energy Pooling Layer

Standard one-dimensional pooling layer: the maximum pooling method is generally adopted, and the convolution calculation method is:

(14) $$p_{i}^{l}(j)=\underset{(j-1)W+1\leq t\leq jW}{\max}\{q_{i}^{l-1}(t)\}$$

where $p_{i}^{l}(j)$ is the corresponding value of l layer of neurons; W is the width of the pooling area; $q_{i}^{l-1}(t)$ is the output value of the *i*th neuron in layer l−1, where t∈[(j−1)W+1,jW].

Root mean square (RMS) is a time-domain statistical feature used to describe signal energy. It has the characteristics of stability and repeatability in the diagnosis indicators. It is an important indicator to judge the operation state of equipment and diagnose component faults. When the index exceeds the normal value, the equipment must have fault or hidden danger. For signal y, the effective value calculation formula is:

(15) $$Y_{rms}=\sqrt{\frac{\sum_{i=1}^{M}y_{i}^{2}}{M}}$$

The multi-channel convolution layer automatically mines the fault feature information of signals with different frequency bands to characterize the fault characteristics of equipment. The energy pooling method based on frequency band energy information extracts the energy features of different frequency bands in the signal. For signal y, the calculation formula of the signal channel energy pooling method is:

(16) $$f(Y)=\sqrt[{p}]{\frac{\sum_{y\in Y}y^{p}}{M}}$$

Combining Equations (15) and (16), when p is infinite, it is equivalent to the maximum pooling operation; when p=1, it is equivalent to the average pooling operation. In this study p=2, it is equivalent to the energy extracted pooling operation. The energy feature extracted from this layer is similar to the RMS feature. It has the characteristics of stability and good repeatability in the diagnostic indicators, which can better judge the operating status of the equipment and diagnose the faults of the components. This method can be used as a frequency band energy index to describe and better identify anomalies in non-stationary signals.

### 3.3. Fully Connected Layer

Standard fully connected layer: rearrange the features extracted by the previous convolution and pooling layers into a column, and the output is:

(17) $$\delta_{i}=f(w_{i}p_{i}+b_{i})$$

where i=1,2,⋯,k; $\delta_{i}$ is the *i*th output, there are k outputs in total; $w_{i}$ and $b_{i}$ are the weights and thresholds of the *i*th neuron respectively; f(x) is the activation function.

Classifier layer: through the softmax classifier, multi-classification tasks can be directly completed.

(18) $$y(i)=\frac{e^{\delta_{i}}}{\sum_{k=1}^{K}e^{\delta_{k}}}$$

where y(i) is the probability of each output, and the sum of all y(i) is 1; K is the number of categories of the multi-classification problem. The output of softmax can be regarded as a probability problem.

## 4. Fault Diagnosis Method of AE Signal of RV Reducer

**Figure 2:**
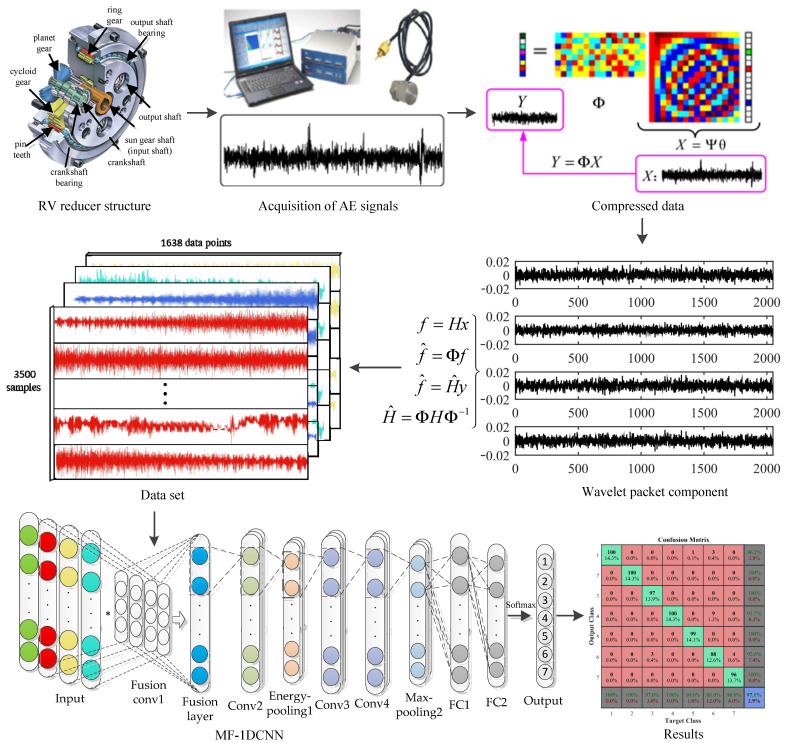
Flow chart.

The structure of an RV reducer is complex and has tightly coupled internal parts. In this study, the fault diagnosis of an RV reducer is based on the AE signal of CS. The combination of CS theory and an MF-CNN model can not only effectively solve the problem caused by AE signal data redundancy, but also adaptively select the frequency band features containing the main feature information to avoid the uncertain influence of a subjective parameter setting on the experimental results. The original signal was compressed and sampled, the amount of data was reduced, and then the compressed signal was decomposed by wavelet packet in compressed domain to obtain the information of each frequency band. The obtained information of each frequency band was used as the input signal of the multi-channel convolutional layer. Then the energy pooling layer was used to mine the energy features of each frequency band. It not only solves the problem of a large amount of AE signal data in fault diagnosis, but also solves the problem of weak fault features of CS data. Finally, the softmax classifier was used to classify and predict RV reducers of different fault types. The basic process of AE signal fault diagnosis of an RV reducer is shown in Figure 2.

In this section, the network structure parameter settings of MF-CNN are shown in Table 1. As the fusion layer of multi-channel data, the first convolution layer uses a wider convolution kernel (64 × 1@4), and the remaining convolution layers use a shorter convolution kernel (32 × 1@1). The wider convolution kernel can not only obtain more frequency band feature information of the signal in the first layer, but also filter the interference of high-frequency noise; while the shorter convolution kernel and the deep network structure can fully mine the hidden fault features of the signal.

In addition, the Dropout function was added to suppress overfitting and improve the generalization ability of the model. In order to solve the problem of gradient disappearance or explosion, batch normalization (BN) was added after each convolution layer. The initial learning rate was set to 0.001 and the decay rate was 0.99. The training process uses small batch learning, and the size was set to 32. All parameters were updated through Backpropagation and the Adam optimization algorithm. In this study, when training the model, the cross-entropy loss function was used to measure the difference distribution between the predicted value and the actual value. The last layer of the network uses the softmax function to output $y_{pred}$, m represents the total number of samples, and $y_{true}$ is the fault category label corresponding to the sample. Therefore, the cross-entropy loss function is defined as:

(19) $$Loss=-\frac{1}{m}\sum_{x}y_{true}\left[\ln y_{{}_{pred}}+(1-y_{true})\ln(1-y_{pred})\right]$$

## 5. Experimental Verification and Result Analysis

### 5.1. Experimental Device and Data Description

**Figure 3:**
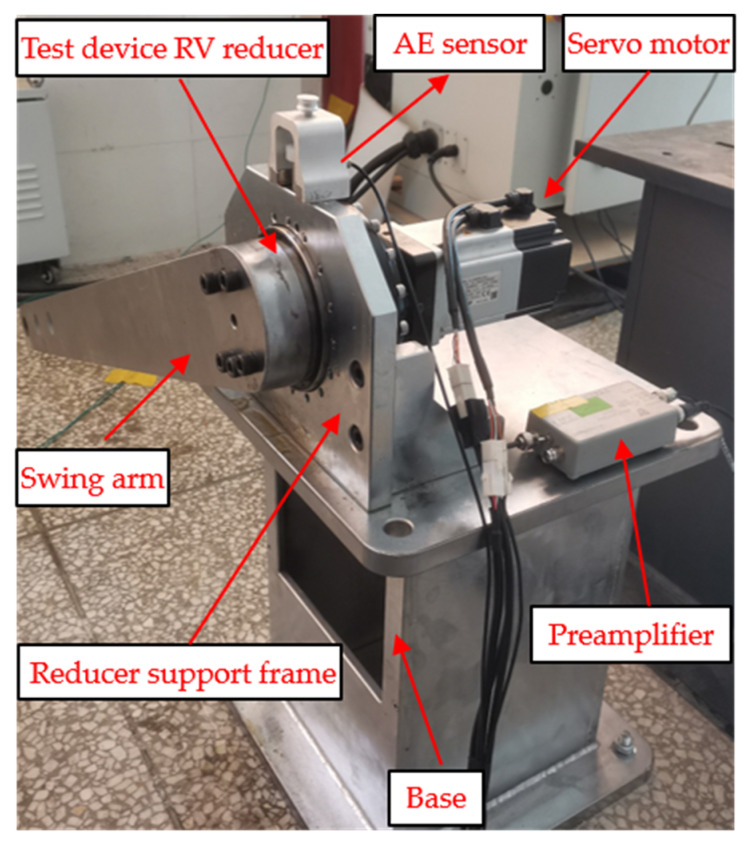
RV reducer failure test bench.

In order to verify the effectiveness of the method proposed in this study, a self-fabricated RV reducer fault test bench was used for experiments. The test bench is shown in Figure 3. The test bench consists of five parts: base, swing arm, servo motor, reducer support frame, and reducer (12 teeth of sun gear and 42 teeth of planet gear).

**Figure 4:**
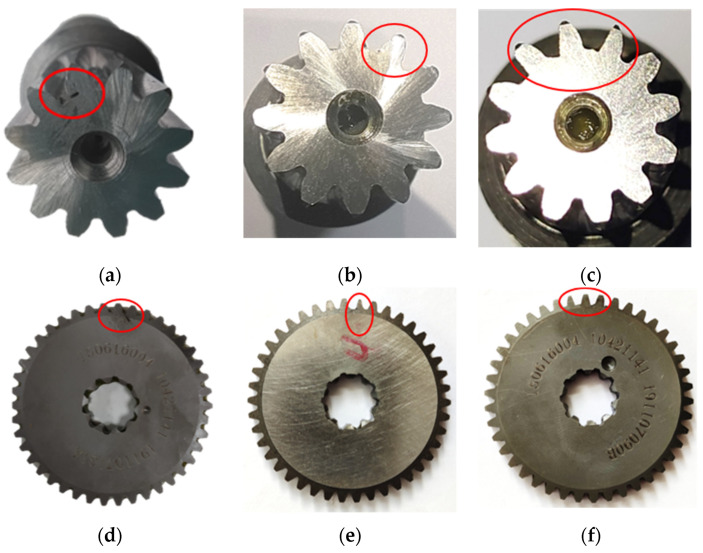
Fault picture. (**a**) Sun gear tooth root crack, (**b**) sun gear single tooth surface wear, (**c**) sun gear multi-tooth surface wear, (**d**) planetary gear tooth root crack, (**e**) planetary gear single tooth surface wear, (**f**) planetary gear multi-tooth surface wear.

The reducer was used as a test device for the fault simulation experiment. The experiment used wire-cut electric discharge technology to cut the sun gear and planetary gears to simulate normal, sun gear root cracks (crack depth is 0.3 mm), planetary gear root crack (crack depth is 0.3 mm), sun gear single tooth surface wear (tooth surface wear size is 0.5 mm), and sun gear multi-tooth surface wear (tooth surface wear dimensions are 0.5 mm, 0.3 mm, and 0.1 mm respectively),planetary gear single tooth surface wear (tooth surface wear size is 0.5 mm), and planetary gear multi-tooth surface wear (tooth surface wear size is 0.5 mm, 0.3 mm, and 0.1 mm respectively), as shown in Figure 4.

In order to reduce the influence of other factors on AE fault signal, the lubricant, load, and other settings met the experimental requirements during the experiment. The output shaft of the RV reducer maintained a reciprocating movement of 90° and the movement speed was 100°/s. The AE acquisition system consists of AE sensor, preamplifier, data acquisition card, host, and display. Using the AE sensor of PAC-R15, its frequency range is 50–400 kHz, and the sampling frequency was set to 1MHz. The preamp was set to 60 dB. The AE sensor was installed above the RV reducer and connected with the reducer support frame by the magnet base. The AE signals collected in the experiment have 8,192,000 data points of each type.

### 5.2. Signal Compression and Reconstruction Verification

**Figure 5:**
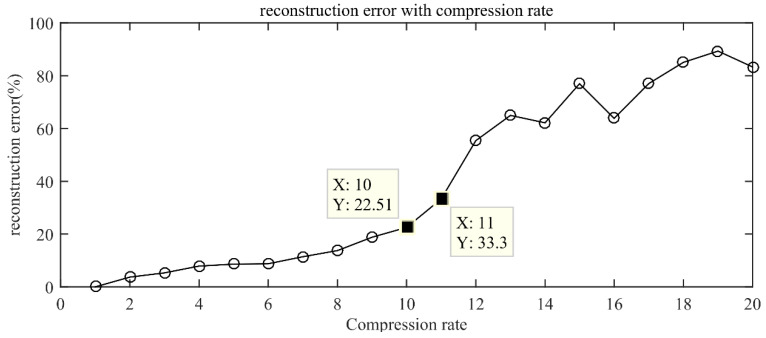
Reconstruction error curve under different compression rates.

The compression rate is a parameter that measures the amount of compressed measurement signal and reflects the degree of compression of the original signal. The larger the compression rate (R = data length of the original signal/data length after compression), the more feature information loss of the original signal in the compressed domain signal. Therefore, it is very important to choose an appropriate compression rate. In the case of retaining key information, the CS method with an appropriate compression rate can significantly reduce the computational cost. In this study, the discrete cosine transform (DCT) method was used to sparse represent the original AE signal, and the orthogonal matching pursuit (OMP) algorithm was used to reconstruct the signal to study the influence of different compression rates on the reconstruction error. The reconstruction error is defined as err=‖x−y‖/‖x‖. The change trend of the reconstruction error was calculated under different compression rates, the compression rate range is 1~20, and the reconstruction error curve under different compression rates were obtained, as shown in Figure 5. The reconstruction error increases with the increase in the compression rate. The larger the reconstruction error, the less feature information in the compressed data. When the compression rate is less than 10, the reconstruction error is less than 22.51%, and if the reconstruction error is less than 25%, it is acceptable for practical applications [15]. The effective key information in the original signal is retained in the compressed measurement data, and the original signal can be reconstructed with a lower reconstruction error. This provides sufficient feature information for the network feature extraction in the compressed domain.

**Figure 6:**
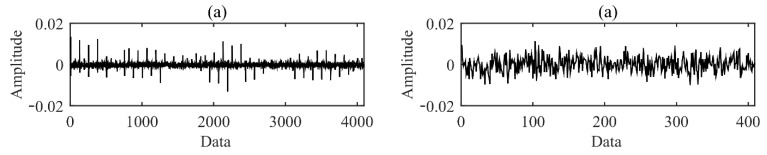

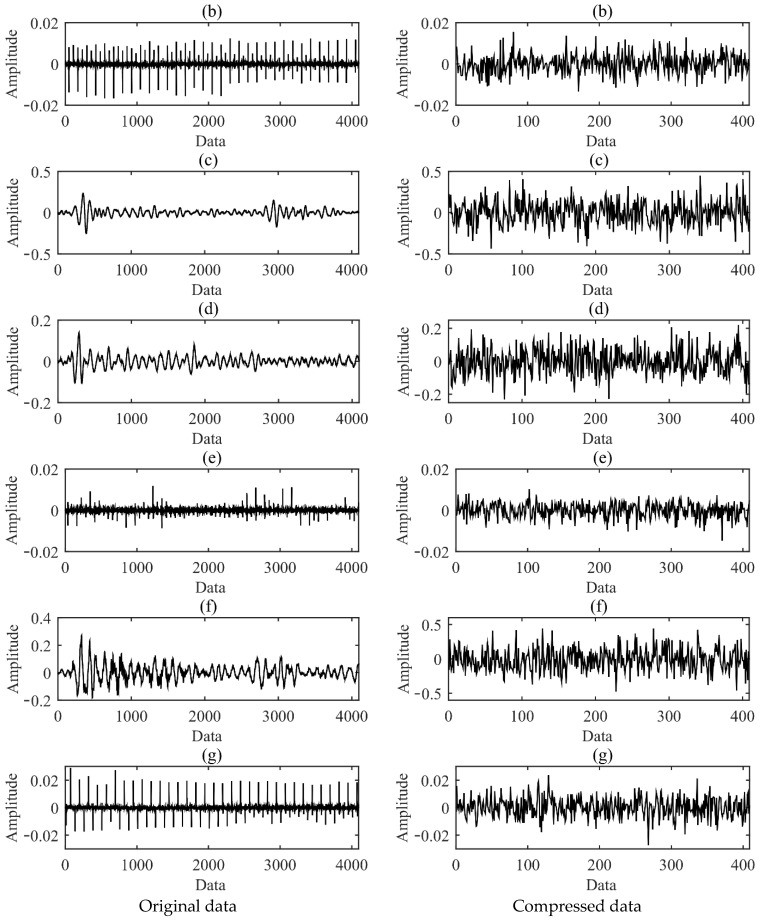
Time waveform. (**a**) Normal, (**b**) sun gear tooth root crack, (**c**) sun gear multi-tooth surface wear, (**d**) sun gear single tooth surface wear, (**e**) planetary gear tooth root crack, (**f**) planetary gear multi-tooth surface wear, (**g**) planetary gear single tooth surface wear.

In this study, the compressed data were obtained by constructing a Gaussian random observation matrix and performing random projection compression on it. According to the error of the reconstruction error curve under different compression rates in Figure 5, a compression rate of 10 times was selected to compress the original signal. The time waveforms of AE signals before and after compression for each fault type are shown in Figure 6. It can be found from Figure 6 that the strength of the impact signal carried by the original signal is different. Compared with the original AE signal, the compressed AE signal presents similar random characteristics in the time domain. This is due to the loss of some time-domain features of the signal during the compression measurement process. At this time, the conventional AE signal analysis methods cannot accurately and effectively carry out feature extraction and classification.

The compressed signal was decomposed by wavelet packet in compressed domain. According to experience, a two-layer wavelet packet decomposition was selected. Each sample signal was decomposed into four component signals with different frequency bands to form four channels as the input of MF-CNN model, which is more comprehensive mine the fault feature information of compressed domain signal. In order to optimize the calculation speed of the model and make the data samples contain more periodic signals, 2^14^ data points of the original data were selected for compression. The compressed data length is 1638. Therefore, 1638 × 4 data points were taken as the sample input length, and each type has 500 groups of data. The size of the data set is 500 × 1638 × 4. According to the proportion setting of 80% and 20%, the data set was divided into training data set and test data set. The training data set contains 400 × 1638 × 4 samples and the test data set contains 100 × 1638 × 4 samples. The sample segmentation information is shown in Table 2.

### 5.3. Experimental Results and Discussion

**Figure 7:**
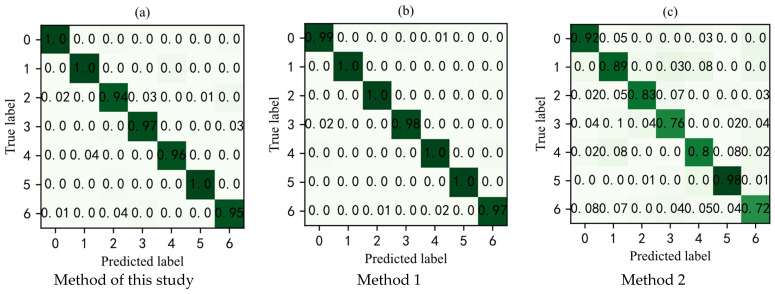
Confusion matrix. 0: normal; 1: sun gear tooth root crack; 2: sun gear multi-tooth surface wear; 3: sun gear single tooth surface wear; 4: planetary gear tooth root crack; 5: planetary gear multi-tooth surface wear; 6: planetary gear single tooth surface wear.

Due to compressed data that shows the randomness of the amplitude, in order to obtain more feature information from the signal, this study used the compressed domain wavelet packet to decompose the compressed signal, and then the component signals of four different frequency bands generated by the signal form four channels as the input of the MF-CNN model. In order to verify the effectiveness of the method in this study, different methods were used for fault diagnosis of RV reducers. Method 1 is that the original signal is directly input into convolutional neural network for fault diagnosis. Method 2 is that the compressed signal directly enters the convolutional neural network for fault diagnosis. Under the condition of the same parameters, the confusion matrix of the predicted label results and the real label results of the test set samples of each method is shown in Figure 7. From Figure 7a, it can be seen that the prediction accuracy of the normal, sun gear tooth root crack, and planetary gear multi-tooth surface wear reached 100%. Figure 7b shows that the prediction accuracy of the failure states of sun gear tooth root crack, sun gear multi-tooth surface wear, planetary gear tooth root crack, and planetary gear multi-tooth surface wear reached 100%. Figure 7c shows that the highest prediction accuracy of the fault state was the planetary gear multi-tooth surface wear, 98% of the planetary gear multi-tooth surface wear was correctly classified, and 1% was wrongly classified as the sun gear multi-tooth surface wear, and the remaining 1% was misclassified as planetary gear single tooth surface wear.

The average predicted correct diagnosis results and training time of each method are shown in Table 3. It can be seen from the table that the diagnostic accuracy of the method proposed in this study can reach 97.43%. It has almost the same effect as method 1 of direct fault diagnosis with the original signal, but it can be greatly improved in efficiency. Method 2 which directly uses compressed signals to diagnose faults can only reach 84.29% accuracy rate, which proves that the method proposed in this study can effectively enhance the ability of signal feature extraction.

**Figure 8:**
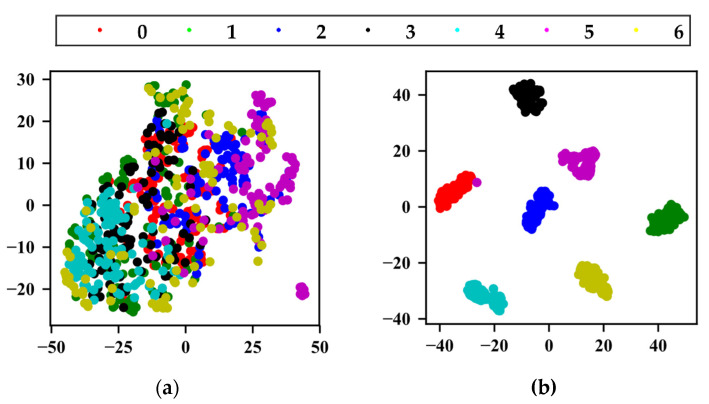

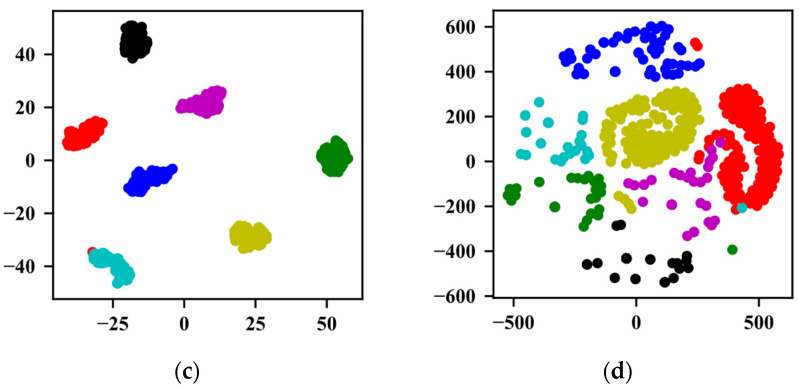
t-SNE dimensionality reduction feature visualization. (**a**) Original data, (**b**) method of this study, (**c**) method 1, (**d**) method 2.

The t-distributed stochastic neighborhood embedding method (t-SNE) proposed by Laurens [30] was used to visualize the features learned by the three different methods. Figure 8a is the dimensionality reduction mapping result of the original data, in which can be seen that the data are disorderly distributed in two-dimensional space; Figure 8b is the mapping result of the last fully connected layer of the method proposed in this study, which showed obvious classification results; Figure 8c is the mapping result of the last fully connected layer of method 1, in which can be seen an obvious classification effect; Figure 8d is the mapping result output from the last fully connected layer of method 2. It can be seen that the basic classification was completed, but the distance between the classes is very small, which does not achieve obvious classification effect.

**Figure 9:**
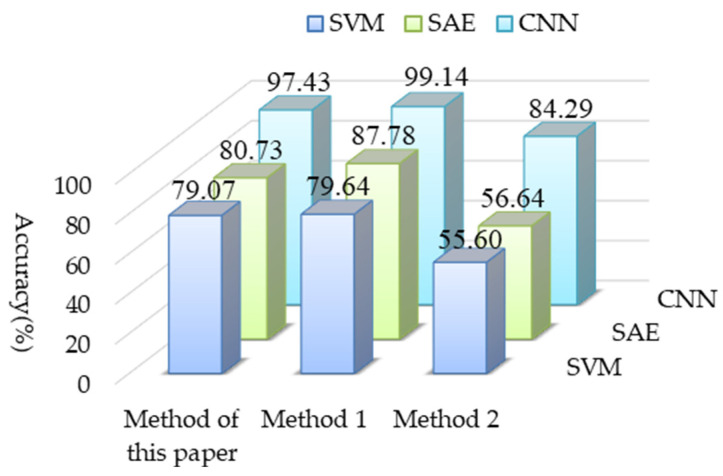
Diagnostic results of different methods.

In order to further illustrate the advantages of the proposed method, it was compared with the sparse auto-encoder (SAE) and support vector machine (SVM) fault diagnosis methods respectively. The radial basis function was selected as the kernel function of SVM. The classification features used by the SVM algorithm are 11 eigenvalues, such as RMS, skew, kurtosis, peak, waveform factor, peak factor, and margin. The diagnosis results are shown in Figure 9. It can be seen from the Figure 9, the diagnosis results of CNN are higher than those of SAE and SVN. This study combines CS theory with MF-CNN. While retaining most of the feature information of the original time-domain signal, and then adaptively selecting the frequency band information through the MF-CNN to enhance the weak fault features of the compressed signal, less data can be used to achieve higher classification accuracy. SAE mainly extracts features from the input data and passes them to the decoder to reconstruct the original data. Aiming at the compressed data of the processing object in this study, all the information of the signal cannot be accurately obtained, resulting in the low fault diagnosis and recognition rate of the network. In addition, the performance of the traditional shallow model of SVM largely depends on subjective artificial feature extraction, so its fault diagnosis accuracy and generalization ability combined with the SVM classifier is low.

## 6. Conclusions

This study presents a diagnosis method of AE signal based on the CS of RV reducers. This method obtains the compressed signal by projecting the time-domain AE signal into the compression domain, which greatly reduces the amount of data redundancy while retaining most of the information, and improves the analysis efficiency. The wavelet packet change matrix in the compression domain was used to decompose the compressed signal and obtain the information of each frequency band. The obtained frequency band information was used as the input signal of multi-channel convolution layer, and then the energy features of each frequency band were mined by energy pooling layer. It not only solves the problem of large amount of AE signal data in fault diagnosis, but also solves the problem of weak fault features of compressed data. The blindness of feature selection of each frequency band component is avoided. Finally, the experimental results show that the accuracy of fault diagnosis can reach 97.43%, which is better than other traditional methods. At the same time, it provides a new diagnosis method for equipment fault diagnosis under modern big data, and carries out fault diagnosis more efficiently under the condition of ensuring high diagnosis accuracy.

## Figures and Tables

**Table 1:** MF-CNN model structure parameters.

Layer	Conv Kernel Size (Length × Width @ Channels)	Activation Function	Output Size (Length × Width @ Channels)
Fusion conv 1	64 × 1@4	ReLU	1638 × 1@1
Conv2	32 × 1@64	ReLU	1638 × 1@64
Energy-pooling 1	2 × 1@64		819 × 1@64
Conv 3	32 × 1@64	ReLU	819 × 1@64
Dropout			
Conv 4	32 × 1@128	ReLU	512 × 1@128
Max-pooling 2	3 × 1@128		273 × 1@128
FC1	216 × 1		216 × 1@1
FC2	64 × 1		64 × 1@1
Softmax	7 × 1		7 × 1@1

**Table 2:** Data set description.

Sample Type	Points	Training Set	Test Set	Mark
Normal	500 × 1638 × 4	400	100	0
Sun gear tooth root crack	500 × 1638 × 4	400	100	1
Sun gear multi-tooth surface wear	500 × 1638 × 4	400	100	2
Sun gear single tooth surface wear	500 × 1638 × 4	400	100	3
Planetary gear tooth root crack	500 × 1638 × 4	400	100	4
Planetary gear multi-tooth surface wear	500 × 1638 × 4	400	100	5
Planetary gear single tooth surface wear	500 × 1638 × 4	400	100	6

**Table 3:** Diagnostic results of different methods.

Method	Accuracy (%)	Training Time (s)
Method of this study	97.43	3500
Method 1	99.14	15,000
Method 2	84.29	2000

## Data Availability

The data presented in this study are available on request from the corresponding author.
